# Trajectories of functional decline and predictors in long-term care settings: a retrospective cohort analysis of Canadian nursing home residents

**DOI:** 10.1093/ageing/afae264

**Published:** 2024-12-03

**Authors:** Bonaventure Amandi Egbujie, Luke Andrew Turcotte, George Heckman, John P Hirdes

**Affiliations:** School of Public Health Sciences, University, 200 University Ave W, Waterloo, ON N2L 3G1,Canada; Department of Health Sciences, Brock University, 1812 Sir Isaac Brock Way, St. Catharines, ON L2S 3A1, Canada; School of Public Health Sciences, University, 200 University Ave W, Waterloo, ON N2L 3G1,Canada; Schlegel-UW Research Institute for Aging, 250 Laurelwood Dr, Waterloo, ON N2J 0E2, Canada; Schlegel-UW Research Institute for Aging, 250 Laurelwood Dr, Waterloo, ON N2J 0E2, Canada

**Keywords:** trajectories, functional decline, aging, inter-resident assessment instrument, long-term care, older people

## Abstract

Decline in the ability to perform activities of daily living (ADL) or ‘*functional decline’* is a major health concern among aging populations. With intervention, ADL decline may be delayed, prevented or reversed. The capacity to anticipate the trajectory of future functional change can enhance care planning and improve outcome for residents.

**Methods:**

This is a 36 months’ retrospective longitudinal analysis of LTC residents in five Canadian provinces. Group-based trajectory modelling (GBTM) was performed to identify distinct trajectories and resident attributes associated with membership of the trajectory groups.

**Results:**

A total of 204 036 LTC residents were included in this study. Their admission mean age was 83.7 years (SD = 8.6), and 63.3% were females. Our model identified four distinct trajectories namely: ‘**Catastrophic decline**’ (*n* = 48 441, 22.7%), ‘**Rapid decline with some recovery**’ (*n* = 27 620, 18.7%), ‘**Progressive decline**’ trajectory (n = 30 287, 14.4%), and the ‘**No/Minimal decline**’ (*n* = 97 688, 47.9%) Residents’ admission ADL Hierarchy score was the single, strongest predictor of functional decline trajectory that residents followed. Residents with ADLH 5–6 OR 0.03 (0.03–0.04) were least likely to follow a catastrophic decline trajectory, while those with ADLH 5–6 OR 39.05 (36/60–41.88) were most likely to follow a minimal or no decline trajectory.

**Conclusion:**

Results of this study further highlight the heterogeneity of health trajectory among residents in LTC setting, re-affirming the need for personalized care. The study shows who among residents would be most at risk for different levels of functional decline.

The study findings provide useful information that would assist both immediate and advanced care planning as well as to forecast care personnel requirements into the future based on total acuity levels of residents.

## Key Points

- Four distinct trajectories of functional decline were identified among these long-term care residents.
- Admission activities of daily living hierarchy score is the strongest predictor of functional decline trajectory.
- Parkinson's disease predisposed to early and rapid decline while dementia predispose to slow progressive decline trajectories.

## Introduction

Decline in the ability to perform activities of daily living (ADL) or ‘*functional decline’* is a major health concern among aging populations, partly because loss of physical function is one of several reasons older adults receive institutionalized care [1–4]. Inability to perform ADLs is particularly important in LTC settings because it is a strong determinant of their resource utilization [5–11]. With intervention, ADL decline may be delayed, prevented or reversed [12–15]. Achieving these outcomes could further be bolstered with accurate forecasting of ADL decline trajectories. However, tools to predict future ADL trajectories in the LTC setting are currently lacking, as existing ones are mostly designed to identify immediate risks [16–18].

Prior to the availability of newer modelling techniques, studies of decline in physical function trajectory mostly represented functional change trajectory using mean population parameter estimates [19, 20]. However, this could mask essential information that are usually embedded within data.

Recent advances in trajectory modelling including group-based modelling techniques [21–23], has availed clinical researchers with additional tools to explore the nuanced trajectory of health measures such as functional decline [24]. The report by the World Health Organization (WHO) that older adults progress through distinct functional trajectories [25], gives further impetus to the need for research into the disaggregated course of functional decline. In recent years, evidence of physical function sub-trajectories in older adults has also grown in the literature. Bimou *et* al. [26], Gill *et al.* [27] and Saito *et* al. [28] provided evidence of distinct functional decline trajectory subgroups among community-dwelling older adults. However, not enough is known about the long-term functional decline trajectory among LTC residents.

Understanding the trajectory of functional decline among LTC residents offers some opportunities and benefits. First, it will allow the early identification of functional limitations and associated potential health challenges. Healthcare professionals could utilize such knowledge to prevent, delay or mitigate the effects of the decline. Some residents could be successfully discharged back to the community with successful intervention. Preventing or delaying further decline may also reduce health system costs. Capacity to anticipate the trajectory of functional change can also inform research efforts to develop new treatments, interventions and technologies to slow down, reverse or adapt to the decline. This knowledge provides a foundation for studying the underlying mechanisms of functional decline, identifying risk factors and exploring potential strategies for intervention. This study seeks to determine the trajectory of functional decline for LTC residents in Canada and to ascertain factors associated with following particular trajectories.

## Methods

### Study design and population

This study is a retrospective longitudinal secondary data analysis of residents admitted into LTC facilities in the Canadian provinces of Alberta, British Columbia, Manitoba, Newfoundland & Labrador and Ontario between January 1st, 2015, and December 31st, 2021. All nursing homes in the five provinces that submitted data to Canadian Institute for Health Information were included in the study. The analyses included each resident’s repeated longitudinal assessments from entry into an LTC facility up to 36 months. The original protocol for this study was developed as part of the PhD thesis proposal for the 1st author.

**Eligibility:** Residents were selected if they: (i) are 60 years or older on admission, (ii) have received at least three assessments beginning with admission, (iii) and are not comatose.

To be selected for the analysis, an LTC resident must have received at least three consecutive assessments from the time of admission. One of the recommendations for implementing the GBTM is that at least three longitudinal data points are used for the model in order to obtain accurate latent trajectories. Usually, LTC residents receive quarterly (3-monthly) assessments, but may be assessed sooner if there is a significant change in their health status. If a resident received 2 quarterly assessments and one other assessment less than three months later because of significant heath change, such residents were also included in the analysis as having received three assessments.

### Data sources

The data for this study was obtained from the Canadian Institute for Health Information’s Continuing Care Reporting System (CCRS). The CCRS contains resident-level data collected through multidimensional health assessments using the interRAI Minimum Data Set 2.0 (MDS 2.0). Full ethical approval for using this data was obtained through University of Waterloo’s Office of Research Ethics (ORE# 30372).

MDS 2.0 assessments are completed by trained assessors within 14 days of patients’ admission to LTC settings and repeated every 90 days after that or sooner in the case of a significant change in health status. The reliability and validity of the MDS assessment items, outcome measures and summary scales are well established [29–33]. The MDS is deployed within a software application, allowing for the generation of scales and Clinical Assessment Protocols, which facilitates care planning at the patient level and program and system-level quality performance assessment. The MDS data do not have missingness due to in-built data field validations and quality check.

### Outcome of interest—operationalizing functional decline

For this study, functional decline was operationally defined as a 1 or more-point increase in the interRAI ADL Hierarchy scale between each assessment and that individual's admission or baseline value. The interRAI ADL Hierarchy scale is a 7-level ordinal measure of functional performance based on a person's ability to complete early (personal hygiene), middle (toileting and locomotion) and late-loss (eating) ADLs [34]. The ADL Hierarchy scale is particularly useful when assessing a system-induced change in ADL, as the summary scales generate wide mean differences when used, with wide standard deviations making them too sensitive and less specific [34]. One-point change in a 7-item Older Americans’ Resource and Services (OARS) ADL scale [range 0–14] was considered to be clinically significant by geriatricians surveyed in Canada [35], while a study by Suijker et al. [36] reported a 0.47 points difference on the KATZ ADL scale [range 0–6] to be a ‘minimally important change’. A 1-point change in the interRAI ADL hierarchy scale is equivalent to a 2.6-point mean change in interRAI short form [range 0–14], similar to the OARS ADL scale. Therefore, a 1-point decline in interRAI ADL hierarchy was deemed to represent a clinically significant change.

### Independent variables

Several independent variables were selected and used for the different phases of analysis performed for this study. These variables were chosen based on previous literature showing their associations with functional decline in institutionalized persons [37–39]. Socio-demographic variables such as age (categorized into <65, 65–74, 75–84 and 85+) and sex were included. Others include the Index of Social Engagement (ISE), Cognitive Performance Scale (CPS) [31], Changes in Health, End Stage Disease and Signs and Symptoms scale [40], acute frailty index [41] (categorized into 0.00–0.20, 0.21–0.30, 0.31–0.40, 0.41+), perceived rehabilitation potential (resident and staff), visual and hearing impairments, number of medications used, chronic conditions such as diabetes, hypertension, congestive heart failure, Alzheimer’s, Parkinson’s, chronic obstructive pulmonary disease, arthritis, falls, unsteady gait, hip fracture, schizophrenia, cancer. A selection of variables representing the number of days a medication was used in the last 7 days by residents for which data was available were also included in the analysis. The medications list includes antipsychotics, antianxiety, antidepressants, hypnotics, diuretics and analgesics. Lastly, we created a binary variable that differentiated COVID-19 period assessments from non-COVID-19 period assessments. This variable was included in the analysis to account for the effect of the pandemic period on trajectory membership. A complete list of all included variables is presented in Appendix 1 shown in the supplementary data section.

### Statistical analysis and modelling

Descriptive analysis was used to summarize the characteristics of the main study participants, showing frequencies and percentages for categorical variables, as well as the mean and standard deviation for continuous variables. Chi-square and Kruskal–Wallis tests were used to check for significant association between two categorical variables depending on the number of categories in the variable and their nature.

### Trajectory modelling

Group-based trajectory modelling (GBTM) [21, 22, 24, 42, 43] was performed to identify distinct trajectories of functional decline among LTC residents.

Change in Bayesian Information Criteria (BIC) value between an alternative (increasingly complex) model and a null (less complex) model was used to evaluate the evidence against the null model [44, 45]. When a more complex model is fitted, and the calculated difference in BIC obtained was greater than 2, the more complex model was selected. The obtained ΔBIC is multiplied by 2 to give the equivalent of the ‘**logged Bayes factor**’ [22, 45]. All models contained the same number of observations when compared.

In addition to using the logged Bayes factor to determine best-fitting models, each trajectory subgroup must have a group membership probability of at least 0.5 and a mean group membership posterior probability of 0.7 for the model to be valid.

Residents who died or were transferred out of the LTC setting before completing 36 months were considered censored or truncated, reflecting a form of attrition. Attrition in the study due to death or discharge before 36 months was accounted for by including a DROPOUT module in the PROC TRAJ model [46]. Haviland, Jones and Nagin demonstrated that adding the dropout module to the model helped to produce unbiased estimates of the model parameters, including trajectory shape and size [47]. By including the dropout module, the model estimates the future trajectory of each resident using either the last assessment value, the assessment value before the last or two assessments before the last. For this study, the assessment before the last provided the best consistent estimate and was chosen for all models.

For each fitted model, the average posterior probability (APP) of the group memberships of 70% or higher and an odd of correct classification (OCC) greater than 5.0 for all obtained groups in a given model shows the fitted model has high assignment accuracy.

Binary logistic regression with backward selection was used to identify resident attributes associated with membership in each of trajectory groups.

All statistical analysis was performed using SAS v9.4 (SAS Institute, Inc., Cary, NC).

## Results

### Baseline characteristics of the primary study sample

The initial study data consisted of 286 124 unique LTC residents, of which 204 036 met the study eligibility criteria and were selected for further analysis. The mean age on admission was 83.7 years (SD = 8.6), and 63.3% were females. Overall, 71.3% had ADL Hierarchy score ≥ 3, 76.6% had impairment in all four ADL Hierarchy items (personal hygiene, toileting, locomotion or eating), while 95.8% had impairment in at least one item. Out of the 204 036 residents, 48 512 (23.8%) died and another 48 334 (23.7%) were transferred out to various destinations before completing 3-year duration of the study. Table 1 displays the admission characteristics of residents by their ADL Hierarchy score on admission.

**Table 1:** Admission characteristics of all residents and by their admission ADL hierarchy scale categories 2015–2021, n = 204 036

Column1.	ADL Hierarchy 0–6 n = 204 036	ADL Hierarchy 0 n = 8405	ADL Hierarchy 1–2 n = 50 263	ADL Hierarchy 3–6 n = 145 368
Variable	% (n)	% (n)	% (n)	% (n)
Age group				
< 65	3.0 (6163)	4.8 (368)	3.0 (1.501)	3.0 (4.294)
65–74	12.7 (25 9462)	16.8 (1408)	12.8 (6407)	12.5 (18 131)
75–84	32.0 (65 373)	33.6 (2826)	33.1 (16 618)	31.6 (45 929)
85+	52.2 (106 554)	45.3 (3803)	51.2 (25 737)	53.0 (77 014)
Sex				
F	63.3 (129 043)	59.2 (4974)	63.7 (32 009)	63.3 (92 060)
M	36.7 (74 993)	40.8 (3431)	36.3 (18 254)	36.7 (53 308)
CPS Scale				
0	9.5 (12 112)	23.0 (1.930)	10.7 (5382)	8.3 (12 112)
1–2	34.6 (70 580)	52.7 (4.350)	42.7 (21 449)	30.8 (44 781)
3–4	45.5 (92 754)	23.4 (1968)	42.4 (21 300)	47.8 (69 486)
5–6	10.4 (21 278)	1.9 (157)	4.2 (2132)	13.1 (18 989)
CHESS Scale				
0	52.9 (107 995)	73.9 (6214)	64.2 (32 254)	47.8 (69 527)
1–2	43.4 (88 570)	25.3 (2129)	34.0 (17 079)	47.7 (69 362)
3+	3.7 (7471)	0.7 (62)	1.8 (930)	4.5 (6479)
Depression Rating Scale (DRS)				
0	51.9 (105 831)	64.7 (5434)	56.1 (28 177)	49.7 (72 220)
1–2	28.3 (57 692)	22.6 (1901)	25.9 (13 023)	29.4 (42 768)
3+	19.8 (40 513)	12.7 (1070)	22.4 (9063)	20.9 (30 380)
Frailty index				
0.01–0.20	5.6 (11 463)	59.3 (4989)	11.4 (5729)	0.5 (745)
0.21–0.30	15.7 (32 111)	33.9 (2849)	36.6 (18 401)	7.5 (10 861)
0.31–0.40	32.2 (65 643)	6.5 (544)	37.9 (18 593)	32.0 (46 506)
> 0.40	46.5 (94 819)	0.3 (23)	15.0 (7540)	60.0 (87 256)
BMI Category				
Underweight	10.0 (20 489)	8.2 (688)	8.5 (4265)	10.7 (15 536)
Normal	44.0 (89 698)	43.3 (3642)	45.3 (3642)	43.6 (63 310)
Overweight	27.3 (55 599)	29.5 (2479)	28.7 (14 438)	26.6 (38 682)
Obese	18.7 (38 250)	19.0 (1596)	17.5 (8814)	19.2 (27 840)
Hearing				
Adequate	59.1 (120 627)	70.3 (5910)	62.5 (31 405)	57.3 (83 312)
Mini Difficulty	26.0 (53 133)	20.7 (1738)	24.9 (12 503)	26.8 (38 892)
Special Situation	13.1 (26 688)	7.5 (635)	11.1 (5572)	14.1 (20 481)
Highly Impaired	1.8 (3588)	1.5 (122)	1.6 (783)	1.9 (2683)
Vision				
Adequate	58.5 (119 438)	73.7 (6197)	65.0 (32,691)	55.4 (80 550)
Impaired	29.1 (59 419)	20.6 (1732)	26.7 (13,400)	30.5 (44 287)
Moderately impaired	6.9 (13 975)	4.0 (337)	5.3 (2669)	7.6 (10 969)
Highly impaired	4.1 (8257)	1.3 (107)	2.2 (1084)	4.9 (7066)
Severely impaired	1.4 (2947)	0.4 (32)	0.8 (419)	1.7 (2496)
Rehabilitation potential	19.1 (39 031)	23.6 (1955)	25.2 (12,656)	16.8 (24 420)
Health Condition & Diagnosis				
Diabetes	25.3 (51 523)	23.2 (1951)	23.6 (11,857)	25.9 (37 715)
Parkinson’s	6.6 (13 417)	3.1 (261)	4.1 (2059)	4.1 (2059)
Unsteady gait	37.7 (76 990)	21.9 (1840)	34.8 (17,472)	39.7 (57 678)
Fall past 30 days	22.4 (45 666)	9.7 (812)	15.2 (7656)	25.6 (37 198)
Stroke	18.0 (36 648)	11.9 (999)	13.4 (6711)	19.9 (28 938)
Hemiplegia/Hemiparesis	3.5 (7166)	0.6 (50)	0.9 (469)	4.6 (6647)
Arthritis	37.3 (76 167)	30.5 (2567)	34.8 (17,502)	38.6 (56 098)
Alzheimer’s disease	14.0 (28 534)	11.7 (983)	15.3 (7695)	13.7 (19 856)
Hypertension	62.7 (128 009)	57.1 (4797)	60.0 (30,169)	64.0 (93 043)
Heart failure	13.0 (19 541)	12.1 (1017)	11.8 (5921)	13.4 (19 541)
Cancer	9.9 (20 168)	9.5 (794)	9.4 (4.724)	10.1 (14 650)
Renal failure	10.7 (21 814)	9.2 (777)	9.9 (4981)	11.1 (16 056)
Impairment in ADLs included in ADL-Hierarchy Scale				
Personal Hygiene	96.6 (197 186)	0.0 (0)	90.0 (17,507)	99.8 (179 679)
Locomotion	84.9 (173 256)	0.0 (0)	42.9 (8349)	91.6 (164 907)
Toileting	93.4 (191 666)	0.0 (0)	68.5 (13,320)	99.0 (178 346)
Eating	82.8 (168 900)	0.0 (0)	54.7 (10,628)	87.9 (158 272)

### Identification of functional decline trajectory subgroups

**Figure 1:**
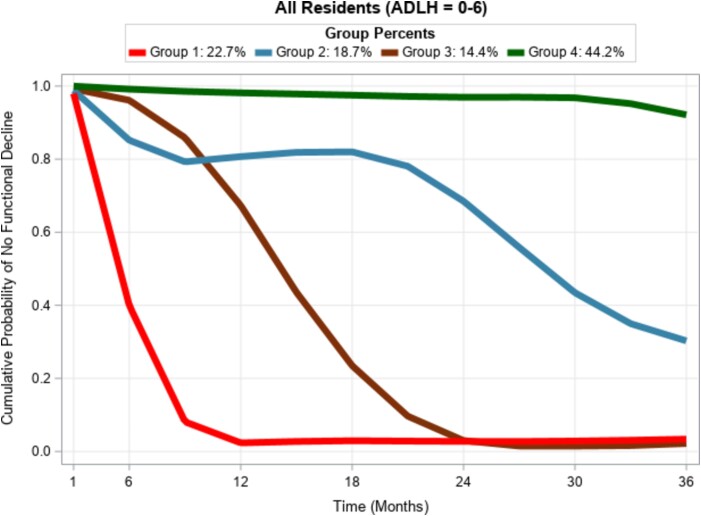
Best Fitting Functional Decline Trajectory Pattern Identified by the GBTM Technique 2015–2021, n = 204 036.

Using GBTM, we identified four distinct trajectories as best fitting for this cohort of LTC residents (Figure 1). The APP of all group assignments was above 0.7, and the OCC was above 5.0 for all groups (See Appendix 2 in the supplementary data section for the full details).

**Figure 2:**
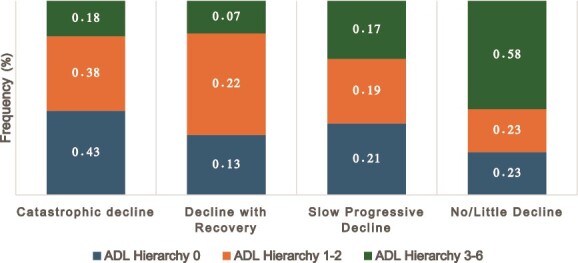
Distribution of Admission ADL Hierarchy Scale within Each Functional Decline Trajectory Groups, n = 204 036.

‘**Catastrophic decline**’ (**Group 1**: *n* = 48 441, 22.7%), in which residents declined precipitously immediately on admission to LTC homes and remained at this lowest functional level until their last assessment (Figure 1). Within the first 90 days of admission, 63.8% of residents who follow this trajectory experienced functional decline. On admission, 43% of residents who follow this trajectory have an ADL Hierarchy Scale of 0, and only 18% had an ADL Hierarchy Scale of 3+ (Figure 2). The mean admission ADHL score was 2.4 and on the next three successive assessments were 3.3, 3.9 and 3.9.

‘**Rapid decline with some recovery**’ (**Group 2**: *n* = 27 620, 18.7%), residents who follow this trajectory experienced immediate steep decline on admission. However, they regain some function soon afterwards (Figure 1). In contrast to the catastrophic decline group, only 62.3% (vs. 98.5%) reported functional decline relative to the admission functional level in their last recorded assessment. For this group, the mean admission ADHL score was 2.9 and on the next three successive assessments were 3.0, 3.0 and 2.9.

‘**Progressive decline**’ trajectory (**Group 3**: n = 30 287, 14.4%)—Residents in this group followed a slower but persistently declining functional trajectory (Figure 1). Only 4.8% of residents in this group declined in physical function within their first 90 days of admission. The mean admission ADHL score for this group was 2.7 and on the next three successive assessments were 2.6, 2.6 and 3.1.

The fourth trajectory, ‘**No/Minimal decline**’ (**Group 4:**  *n* = 97 688, 47.9%)—Residents here had little to no further decline following admission into LTC homes but remained at or near their admission functional level for the three-year duration of the study (Figure 1). Only 0.54% of residents in this group experienced functional decline within 90 days of admission. By their last recorded assessment, only 17.5% declined further in function relative to admission. Compared to the catastrophic (18%) and slow progressive (17%) decline groups, 58% of residents in this group had ADL Hierarchy Scale 3+ impairment on admission (Figure 2).

Residents who followed different functional decline trajectories differed in their baseline physical function status. Those who follow a No/Minimal decline trajectory were more likely to have a loss in the four ADL hierarchy items and late loss ADL (eating), while those who follow the catastrophic decline trajectory were more likely to have no ADL item loss compared to the rest (See Appendix 3 and 4 in the supplementary data section for the full details).

### Multivariable analysis

Table 2 displays the predictors of trajectory group membership obtained using multiple binary logistic regression.

**Table 2:** Adjusted odds of membership by functional decline trajectory group 2015–2021, n = 204,036

		*Catastrophic decline* (n = 48,441)	*Rapid decline with recovery* (n = 27,620)	*Slow progressive decline* (n = 30,287)	***No/Minimal** decline.* (n = 97,688)
Variable	**Category**	OR (95% CI)	OR (95% CI)	OR (95% CI)	OR (95% CI)
Demography					
Age group	<65				
	65–74	1.24 (1.16–1.34)	0.95 (0.88–1.03)	1.15 (1.05–1.25)	0.83 (0.78–0.88)
	75–84	1.35 (1.26–1.45)	0.97 (0.89–1.04)	1.16 (1.07–1.27)	0.78 (0.73–0.83)
	85+	1.42 (1.33–1.53)	0.91 (0.88–0.94)	1.16 (1.07–1.27)	0.79 (0.75–0.84)
Sex	F				
	M	^a^	0.91 (0.88–0.94)	0.96 (0.93–0.98)	1.08 (1.06–1.10)
Clinical Summary Scale					
ADL Hierarchy Scale	0				
	1–2	0.81 (0.76–0.85)	0.93 (0.87–1.00)	1.07 (1.00–1.13)	1.35 (1.27–1.44)
	3–4	0.22 (0.21–0.23)	1.03 (0.96–1.10)	0.79 (0.75–0.84)	5.43 (5.10–5.77)
	5–6	0.033 (0.03–0.04)	0.33 (0.31–0.36)	0.21 (0.20–0.23)	39.15 (36.60–41.88)
CPS Scale	0				
	1–2	0.81 (0.78–0.85)	0.92 (0.88–0.97)	1.28 (1.22–1.35)	1.09 (1.05–1.13)
	3–4	0.95 (0.91–0.99)	0.87 (0.83–0.92)	1.38 (1.31–1.45)	0.96 (0.93–1.00)
	5–6	1.28 (1.21–1.35)	0.85 (0.80–0.91)	1.39 (1.30–1.48)	0.79 (0.75–0.83)
CHESS Scale	0				
	1–2	1.11 (1.08–1.13)	0.94 (0.91–0.96)	0.94 (0.91–0.96)	1.00 (0.98–1.02)
	3+	1.04 (0.98–1.13)	0.92 (0.85–0.99)	0.84 (0.78–0.90)	1.13 (1.06–1.19)
Depression Rating Scale (DRS)	0				
	1–2	1.02 (0.99–1.050	0.97 (0.94–1.00)	1.03 (1.00–1.06)	0.98 (0.96–1.00)
	3+	1.04 (1.01–1.07)	0.94 (0.91–0.98)	1.05 (1.01–1.09)	0.96 (0.94–0.99)
Social Engagement Scale	0				
	1	^a^	^a^	^a^	1.08 (1.02–1.13)
	2	^a^	^a^	^a^	1.14 (1.09–1.20)
	3	^a^	^a^	^a^	1.20 (1.14–1.25)
	4	^a^	^a^	^a^	1.33 (1.27–1.39)
	5	^a^	^a^	^a^	1.34 (1.27–1.41)
	6	^a^	^a^	^a^	1.39 (1.31–1.47)
Clinical items					
BMI Category	Normal				
	Underweight	1.01 (0.97–1.05)	0.91 (0.88–0.97)	0.91 (0.87–0.96)	1.09 (1.05–1.13)
	Overweight	1.04 (1.01–1.07)	1.01 (0.98–1.04)	1.04 (1.01–1.07)	0.94 (0.92–0.96)
	Obese	1.14 (1.11–1.18)	1.02 (0.98–1.06)	1.00 (0.96–1.03)	0.88 (0.86–0.91)
					
Hearing	Adequate				
	Mini Difficulty	0.97 (0.95–1.00)	0.99 (0.96–1.02)	0.98 (0.95–1.01)	1.05 (1.02–1.07)
	Special Situation	0.95 (0.92–0.99)	0.95 (0.91–0.99)	0.96 (0.92 = 3–1.00)	1.10 (1.07–1.14)
	Highly Impaired	0.90 (0.83–0.99)	1.13 (1.02–1.25)	0.88 (0.80–0.98)	1.10 (1.01–1.18)
Vision	Adequate				
	Impaired	1.06 (1.03–1,09)	^a^	^a^	0.95 (0.93–0.97)
	Moderately impaired	1.11 (1.06–1.16)	^a^	^a^	0.90 (0.86–0.93)
	Highly impaired	1.28 (1.21–1.36)	^a^	^a^	0.80 (0.76–0.85)
	Severely impaired	1.39 (1.26–1.53ami)	^a^	^a^	0.77 (0.71–0.84)
Rehab Potential	Yes	0.93 (0.90–0.96)	^a^	0.96 (0.93–0.99)	1.11 (1.09–1.14)
Unsteady gait	Yes	1.05 (1.03–1.08)	0.96 (0.94–0.99)	^a^	0.97 (0.95–0.99)
Fall past 30 days	Yes	1.22 (1.19–1.26)	0.93 (0.90–0.96)	0.96 (0.93–0.99)	0.92 (0.90–0.94)
Hip fracture	Yes	0.90 (0.85–0.95)	^a^	0.82 (0.79–0.87)	1.19 (1.14–1.24)
Health conditions					
Diabetes	Yes	1.04 (1.01–1.07)	^a^	^a^	^a^
Congestive heart failure	Yes	^a^	0.91 (0.87–0.95)	0.93 (0.89–0.97)	1.07 (1.04–1.11)
Osteoporosis	Yes	^a^	^a^	1.03 (1.00–1.07)	^a^
ALS	Yes	2.28 (1.76–2.94)	0.51 (0.34–0.76)	1.47 (1.08–1.99)	0.56 (0.44–0.72)
Alzheimer’s	Yes	1.13 (1.09–1.17)	^a^	1.23 (1.19–1.28)	0.80 (0.78–0.83)
Stroke	Yes	^a^	^a^	^a^	^a^
Dementia	Yes	1.04 (1.01–1.07)	^a^	1.14 (1.11–1.18)	0.91 (0.89–0.94)
Hemiplegia/Hemiparesis	Yes	^a^	^a^	0.76 (0.70–0.83)	1.11 (1.05–1.17)
Huntington’s Chorea	Yes	1.56 (1.12–2.18)	^a^	1.47 (1.03–2.14)	0.56 (0.40–0.76)
MS	Yes	^a^	^a^	0.74 (0.62–0.90)	^a^
Parkinson’s	Yes	1.30 (1.24–1.36)	^a^	1.16 (1.11–1.23)	0.74 (0.71–0.77)
TIA	Yes	1.08 (1.03–1.14)	1.07 (1.01–1.13)	^a^	0.89 (0.85–0.94)

*(continued)*

**Figure 3:**
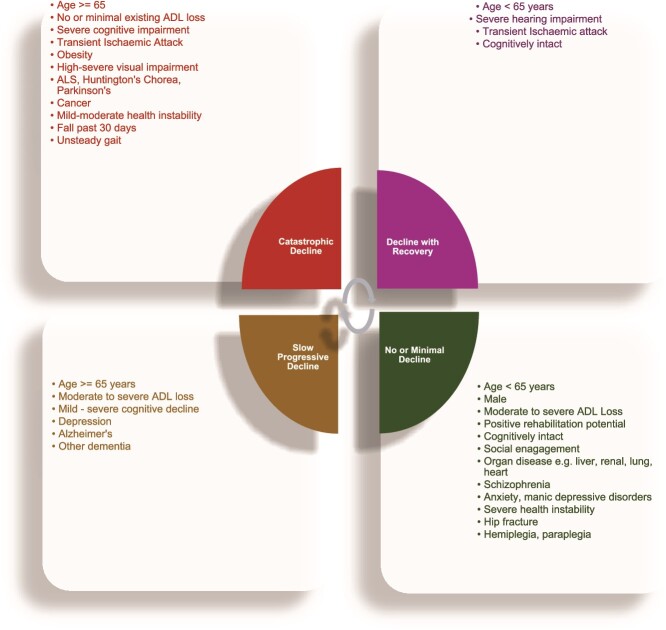
Predicted Membership Profile for the Different Functional Decline Trajectories, n = 204 036.

Admission ADL Hierarchy Scale was the strongest independent predictor of trajectory group membership. Residents without impairment on admission were most likely to follow a catastrophic decline trajectory compared to those with mild [ADLH 1–2] (OR 0.80 95% CI 0.76–0.85), moderate [ADLH 3–4] (OR 0.22 95% CI 0.21–0.23) or severe impairments [ADLH 5–6] (OR 0.033 95% CI 0.031–0.035). Likewise, residents admitted with neurodegenerative conditions such as Amyotrophic Lateral Sclerosis (ALS) (OR 2.23 95% CI 1.73–2.88), Huntington’s chorea (OR 1.52 95% CI 1.09–2.12) and Parkinson’s disease (1.28 95% CI 1.23–1.34) were also more likely to follow a catastrophic decline trajectory. Being cognitively impaired, having Alzheimer’s disease (OR 1.23 95% CI 1.18–1.28) or other dementia (OR 1.14 95% CI 1.11–1.18) also predicted a higher likelihood of following a slow progressive decline trajectory. A summarized profile of trajectory membership based on multivariable regression analysis is shown in Figure 3.

**Table 2:** Continued

		*Catastrophic decline* (n = 48,441)	*Rapid decline with recovery* (n = 27,620)	*Slow progressive decline* (n = 30,287)	***No/Minimal** decline.* (n = 97,688)
TBI	Yes	^a^	^a^	^a^	1.16 (1.05–1.28)
Anxiety disorder	Yes	0.89 (0.86–0.92)	^a^	^a^	1.10 (1.07–1.14)
Manic depressive disorder	Yes	^a^	^a^	^a^	1.10 (1.02–1.19)
Schizophrenia	Yes	0.61 (0.56–0.67)	1.17 (1.07–1.28)	0.85 (0.77–0.94)	1.46 (1.35–1.57)
Emphysema	Yes	0.94 (0.90–0.97)	0.92 (0.89–0.97)	^a^	1.15 (1.11–1.18)
Cancer	Yes	1.07 (1.03–1.11)	0.93 (0.89–0.95)	^a^	^a^
Liver disease	Yes	^a^	^a^	0.93 (0.78–0.99)	1.18 (1.08–1.29)
Renal failure	Yes	^a^	0.91 (0.88–0.96)	^a^	1.06 (1.02–1.09)
Medications (Number of days used in the last 7 days					
Antipsychotic		1.02 (1.01–1.02)	^a^	^a^	0.99 (0.98–0.99)
Antianxiety		^a^	1.01 (1.00–1.02)	0.99 (0.98–0.99)	1.01 (1.00–1.01)
Antidepressant		1.00 (1.00–1.00)	^a^	1.01 (1.00–1.01)	0.99 (0.99–0.99)
Hypnotics		^a^	^a^	^a^	^a^
Diuretic		1.00 (1.00–1.01)	^a^	1.01 (1.00–1.01)	0.99 (0.99–0.99)
Analgesics		^a^	^a^	0.99 (0.98–0.99)	^a^
COVID-19 period	Yes	1.05 (1.02–1.08)	0.46 (0.44–0.47)	0.55 (0.53–0.57)	1.94 (1.89–1.99)

^a^No significant association found (OR was not explicitly written because the model output excluded ORs for non-significant predictors; TIA = Transient Ischemic Attack; TBI = Traumatic Brain Injury; ALS = Amyotrophic Lateral Sclerosis; MS = Multiple Sclerosis

## Discussion

We aimed to identify the trajectories of functional decline for residents in LTC settings, and secondarily, determine the individual-level factors that predict membership of the identified trajectory groups. Our modelling results show that LTC residents progress through four distinct functional decline trajectories upon placement in the setting namely: ‘catastrophic’, rapid decline with recovery”, ‘slow progressive’ and ‘no/minimal’ decline. The profile of members in each trajectory differed substantially, suggesting that individual-level factors determine how residents decline in function over time. Residents’ admission ADL Hierarchy score was the strongest, single predictor of functional decline trajectory that residents followed.

Our modelling result is consistent with prior studies on the trajectories of functional decline among nursing home residents, which have identified four distinct trajectories of functional decline [48, 49]. However, this was the first study to utilize a large population-based administrative dataset to model trajectory of functional decline among nursing home residents in Canada.

Residents who had better baseline ADL scores were more likely to follow the catastrophic decline trajectory compared to those with worse ADL score, who themselves were more likely to follow the no/minimal decline trajectory. One likely explanation for this difference in trajectory membership is the phenomenon of ceiling effect [50–52], where residents with worse (higher) ADL scores have little or no room for further decline.

Profiling analysis also showed that residents who had some form of neurodegenerative conditions such as Parkinson’s disease, ALS and Huntington’s Chorea were more likely to experience a catastrophic decline trajectory irrespective of their baseline ADL score (Table 2). The effect of neurodegenerative conditions such as Parkinson’s disease on functional decline is well established [53–56], with suggestions that it affects both motor and cognitive function. Some of these conditions have no meaningful treatment, while others do (Parkinson’s), with Implications on neurological support for some, and advance care planning for others.

Findings from this study further showed an unexpected separation between the decline trajectory associated with neurodegenerative conditions where motor function loss is the predominant initial symptom (ALS, Parkinson’s and Huntington’s disease) and those where cognitive impairment is the predominant primary symptom (Alzheimer’s disease and other dementia). Except for those who had severe cognitive decline, residents in the latter group with predominantly poor cognitive function (Alzheimer’s disease and other dementia), followed a slow progressive functional decline trajectory. Furthermore, although previous studies show that cognitive impairment is associated with worse functional ability for residents in nursing homes [57], this study showed that on admission, residents with intact cognitive function, as well as those with severe cognitive impairment, were more likely to experience catastrophic functional decline compared to those with mild to moderate cognitive impairment. As shown by the multivariable regression output (Table 2), residents who have other predisposing conditions such as severe visual impairment, cancer, mild to moderate health instability, obesity, recent falls were equally also prone to catastrophic decline even though their cognitive function is intact.

This study also identified a few positive markers of potential to recover in function upon LTC placement. Recovery of physical function is crucial for LTC residents since it could facilitate their return to the community. Cognitively intact residents, those who had severe visual and hearing impairment, transient ischemic attack and those diagnosed with schizophrenia were all likely to recover in physical function. Except for cognition, a common attribute between the markers is that they are treatable or modifiable, suggesting that residents who are identified early could be supported to improve in function. It could be that these types of residents fared well because of the LTC care they have received but could also be an indicator that they can be discharged back home with appropriate home care support.

This study provides relevant clinical information about residents’ functional decline trajectory that is useful in many ways. Clinicians can utilize the trajectory groups' information to set treatment goals or expectations for residents with specific health conditions. Knowing which trajectory residents with particular health conditions would likely follow could guide clinicians on what care planning would be most appropriate for such individuals and the timing of any functional improvement intervention. For clients at risk of catastrophic decline, early intervention would be appropriate to delay or prevent such a decline. Similarly, trajectory information obtained by our modelling and the associated profiles could be utilized by care providers for advanced care planning (ACP). Currently ACP is focused generally on cardio-pulmonary resuscitations, death and somewhat on hospitalization, which can accelerate functional decline. Understanding the risk of further decline is helpful for residents when contemplating procedures that could accelerate their functional demise.

Clinicians can also utilize such information for patient education, informing clients of the likely course of their health and what possible action would be helpful. Informed residents are more likely to be engaged with their management plan, which could optimize health outcomes.

### Limitations

Short-stay residents with less than three assessments were excluded from the analysis, which could introduce bias into the sample. For this reason, it will not be appropriate to generalize the findings to all LTC residents. However, because this study focused on the longitudinal changes in ADL among long-stay residents, it is reasonable to assume that the findings would not be biased for the target resident types. The trajectories identified in this study cannot be generalized beyond the Canadian long-stay LTC residents, since data utilized was specific to this setting. However, the utilization of large population-based data allows us to reasonably generalize the findings to long-stay residents in the five provinces that provide data for the analysis.

## Conclusion

Personalized care is necessary to achieve optimal health outcomes such as physical function for LTC residents. Optimizing physical function among residents will benefit from the utilization of nuanced person-level trajectory information such as provided in this study, for care planning and delivery. Results of this study further highlight the heterogeneity of health trajectory among residents in LTC setting, re-affirming the need for personalized care. The study shows who among residents would be most at risk for different levels of functional decline.

For clinicians, the study findings provide useful information about potential trajectory of residents’ functional levels based on their admission profile. Such information would assist both immediate and advanced care planning. For care administrators, it makes available information that could be used to forecast personnel requirements based on total acuity levels of residents into the future, and ultimately used for staff or other resource planning.

For the findings to be useful however, the evidence from this study would need to be transformed into decision-making tools. Future studies should also investigate how the trajectory of functional decline relates with resource requirement and utilization in LTC settings. Such a study could provide evidence that would be used to optimize resource allocation to LTC facilities.

## Supplementary Material

aa-24-0584-File002_afae264
