# Supplementary material for: Trajectories of functional decline and predictors in long-term care settings: a retrospective cohort analysis of Canadian nursing home residents
Source: Age Ageing. 2024 Dec 3;53(12):afae264. doi: 10.1093/ageing/afae264 (PMC11645752; doi:10.1093/ageing/afae264)
Supplement: aa-24-0584-File002_afae264 [file aa-24-0584-file002_afae264.docx]

Trajectories of Functional Decline and Predictors in Long-Term Care Settings: A Retrospective Cohort Analysis of Canadian Nursing Home Residents.

Contents

[**Appendix 1:** Complete list of all included independent variables. 1](#_Toc181611917)

[**Appendix 2:** Diagnostic Tests Scores for Trajectory Groups Showing APP and OCC. 3](#_Toc181611918)

[**Appendix 3**: Percentage Distribution of Count of ADL Item Loss by Trajectory Group. 4](#_Toc181611919)

[**Appendix 4:** Distribution of ADL item loss by trajectory subgroups 2015 – 2020, n = 204,036. 4](#_Toc181611920)

### **Appendix 1:** Complete list of all included independent variables.

|  | Variable | Description | Range and Levels | Reference |
| --- | --- | --- | --- | --- |
| 1 | ADL Hierarchy Scale | A measure of functional performance in 4 activities of daily living from early to late loss (hygiene, locomotion, toilet use, eating) | 0 - 6  independent to dependent | Morris et al., 1999; Morris et al., 2013 |
| 2 | Acute Frailty Index | Measures the proportion of assessed deficit present | 0.0 – 1.0  robust to frail  Collapsed into Frailty index categories:  [0.0-0.2, 0.21-0.30, 0.31-0.40, >0.40] | Hubbard et al, 2015 |
| 3 | Cognitive performance scale | Measures cognition. | 0 – 6  intact to severe impairment  [Collapsed into **0, 1-2, 3+]** | Morris et al., 2016 |
| 4 | Depression rating scale | Measures depression | 0 – 6  No symptoms to severe symptoms  [Collapsed into **0, 1-2, 3+]** | Burrows et al. 2000; InterRAI 2015 |
| 5 | CHESS scale | Measures medical complexity and instability | 0 – 5  most stable to most unstable  [Collapsed into **0, 1-2, 3+]** | Hirdes et al., 2003; Hirdes et al., 2014 |
| 6 | SOCENG Scale |  |  |  |
| 7 | Resource Utilization Group (RUG) Categorization | Measures and classifies residents according to the level of care required | 1 - Special Rehabilitation  2 - Extensive Services  3 - Special Care  4 - Clinically Complex  5 - Impaired Cognition  6 - Behavioral Problems  7 - Physical Functions Reduced | Fries et al, 1994 |
| 8 | Visual impairment | Measure ability to see in adequate light (with glasses if used) | 0 - 4  Adequate to severe visual impairment | MDS 2.0 Manual |
| 9 | Making self-understood | Measures the ability to express information content, however able | 0 – 3  understood to rarely or never understood | MDS 2.0 Manual |
| 10 | Ability to understand others | Measures the ability to understand verbal information content, however able | 0 – 3  understands to rarely or never understands | MDS 2.0 Manual |
| 11 | Rehabilitation potential | A composite score for where:  Care Staff or resident believes self to be capable of increased independence in at least some ADLs.  Measures ADL functional rehabilitation potential. | 0,1  neither patient nor care staff believes to both believe the patient is capable of increased independence in at least one ADL |  |
| 12 | Respite Care |  | Yes/No | MDS 2.0 Manual |
| 13 | Full bed rails |  | Yes/No | MDS 2.0 Manual |
| 14 | Fell in past 30 days |  | Yes/No | MDS 2.0 Manual |
| 15 | Hip fracture past 180 days |  | Yes/No | MDS 2.0 Manual |
| 16 | Hypertension |  | Yes/No | MDS 2.0 Manual |
| 17 | Osteoporosis |  | Yes/No | MDS 2.0 Manual |
| 18 | Alzheimer’s |  | Yes/No | MDS 2.0 Manual |
| 19 | Parkinson’s |  | Yes/No | MDS 2.0 Manual |
| 20 | Quadriplegia |  | Yes/No | MDS 2.0 Manual |
| 21 | Traumatic brain injury |  | Yes/No | MDS 2.0 Manual |
| 22 | Anxiety |  | Yes/No | MDS 2.0 Manual |
| 23 | Hypotension |  | Yes/No | MDS 2.0 Manual |
| 24 | Unsteady gait |  | Yes/No | MDS 2.0 Manual |
| 25 | Personal Hygiene* | Measures how patient moves to and from lying position, turns from side to side, and positions body while in bed | 0 –4, 8  independent to total dependence, activity did not occur | MDS 2.0 Manual |
| 26 | Eating* | Measures how resident moves between surfaces-to and from: bed, chair, wheelchair, standing position (Excluding to and from bath and toilet) | 0 –4, 8  independent to total dependence, activity did not occur | MDS 2.0 Manual |
| 27 | Walk in room* | Measures how resident walks between locations in own room | 0 –4, 8  independent to total dependence, activity did not occur | MDS 2.0 Manual |
| 28 | Toilet use* | Measures how resident uses the toilet room (or commode, bedpan, urinal); transfers on/off toilet, cleanses, changes pad, manages ostomy or catheter. Adjusts clothes | 0 –4, 8  independent to total dependence, activity did not occur | MDS 2.0 Manual |
|  | *** Not in the final model, but sub-analyzed** | | | |

### **Appendix 2:** Diagnostic Tests Scores for Trajectory Groups Showing APP and OCC.

| Category of residents | Metric | Catastrophic decline | Progressive decline | Rapid decline with recovery | No/Minimal decline |
| --- | --- | --- | --- | --- | --- |
| ADL Hierarchy  0-6 | APP | 0.91 | 0.87 | 0.81 | 0.88 |
|  | Group Probability | 0.23 | 0.19 | 0.14 | 0.44 |
|  | OCC | 33.85 | 28.53 | 26.19 | 9.33 |

### **Appendix 3**: Percentage Distribution of Count of ADL Item Loss by Trajectory Group.

### **Appendix 4:** Distribution of ADL item loss by trajectory subgroups 2015 – 2020, n = 204,036.
